# Treatment patterns and comorbid burden of patients newly diagnosed with multiple sclerosis in the United States

**DOI:** 10.1186/s12883-020-01882-2

**Published:** 2020-08-11

**Authors:** David M. Kern, M. Soledad Cepeda

**Affiliations:** Janssen Research and Development, 1125 Trenton Harbourton Rd, Titusville, NJ 08560 USA

**Keywords:** Multiple sclerosis, Disease modifying therapy, Treatment patterns, Comorbidity, Administrative claims

## Abstract

**Background:**

The treatment landscape for multiple sclerosis (MS) is quickly evolving. Understanding real-world treatment patterns of patients is necessary to identifying potential gaps in care.

**Methods:**

Patients with incident MS were identified from a large national claims database during 1/1/2014–6/30/2019. Patients had ≥2 diagnoses for MS or an inpatient hospitalization with a primary diagnosis of MS. Patients were required to have enrollment in the database ≥1 year prior to and ≥ 1 year following their first MS diagnosis. Treatment sequences were captured for all available disease modifying therapies (DMTs) during all available follow-up. Presence of comorbid conditions were captured during the one year prior to and following (and including) the index date; absolute change in prevalence from the pre- to post-index periods was calculated.

**Results:**

We identified 5691 patients with incident MS. Common comorbidities included physical symptoms (e.g., pain, weakness, fatigue), mental health conditions (anxiety, depression), and cardiovascular/metabolic conditions (hypertension, hyperlipidemia, diabetes, obesity). Just 1994 (35.0%) of patients received a DMT at any time during follow-up. Of those receiving a DMT, 28.2% went on to receive a second line of therapy, 5.8% received a third, and just 0.9% went on to a fourth line. Use of more than one DMT concomitantly occurred in just 1.8% of all treated patients. Glatiramer and dimethyl fumarate were by far the most common first-line treatments received accounting for nearly 62% of patients receiving a DMT.

**Conclusion:**

Approximately two-thirds of patients newly diagnosed with MS did not receive a DMT and the disease is accompanied by a significant comorbid burden.

## Background

Multiple sclerosis is an inflammatory autoimmune disorder of the central nervous system (CNS) and the most common cause of progressive neurological disability in young adults [1]. This chronic demyelinating disease is characterized by a varied clinical expression with an unpredictable course and a variable prognosis. This disease has important personal, social, and financial consequences for patients, their families, and health care systems. The etiology of MS is still unknown, but it is widely accepted that it is an immune mediated, demyelinating process precipitated by unknown environmental factors in genetically susceptible people [2–4].

The disease is typically divided into two partially overlapping phases. After an initial phase of relapsing-remitting multiple sclerosis (RRMS) patients may transition to secondary progressive MS (SPMS), characterized by continuous worsening of symptoms, such as fatigue or cognitive impairment [5]. Currently available disease-modifying therapies (DMTs) address the RRMS phase of MS and are less efficacious in the progressive phase. DMTs work by controlling, segregating, blocking, or depleting disease-causing autoimmune cells, thus limiting their ability to enter and damage the CNS [6], with the goal of reducing disease activity that contributes to long-term disability [7]. In RRMS, the major aims of treatment are to reduce relapses and prevent permanent disability accumulation.

There are currently more than a dozen approved DMTs for treatment of RRMS [8] with different efficacy and safety profiles, including injectable interferons (interferons β-1a and β-1b) and glatiramer acetate, oral therapies such S1P receptor modulators (fingolimod, siponimod, and ozanimod), dimethyl fumarate (DMF), and teriflunomide, and intravenous monoclonal antibodies. With the availability of several first-line (e.g., interferon β, glatiramer acetate) and second-line (e.g., natalizumab, alemtuzumab) therapies, the choice of initial MS therapy and the switch from one therapy to another is based on considerations of efficacy, safety, tolerability, and convenience of treatment administration, and is quickly evolving.

Prior research has provided groundwork for quantifying the general landscape of DMT use including an examination of the proportion of patients diagnosed with MS who receive a DMT [9] and those that receive combination therapy [10]. Much of the research on DMTs has dealt with persistence to therapy, including the first DMT received [11–13] and those examining specific methods of administration such as injectable use [14] and use of oral therapies [15]. Others [16] have examined how use of DMTs has changed over a 10 year period. However, there is a lack of real-world evidence on more detailed treatment patterns in recent years, and an absence of research that looks beyond the first treatment change.

This study examines all available follow-up of patients newly diagnosed with MS in the last 5 years and captures treatment patterns through the first four lines of therapy received. Understanding current landscape will provide a meaningful reference to understand the impact of multiple newly approved medications coming to market.

## Methods

### Patient identification

We identified patients diagnosed with MS from a large national insurance claims database during 1/1/2014 through 6/30/2019. We chose the most recent five and one-half years of data in order to capture a snapshot of current treatment practices rather than historical treatment practices which may no longer be relevant. More detail about the database is found in the ‘Data Source’ section below. Newly diagnosed multiple sclerosis was defined according to the presence of at least two claims with a diagnosis code for MS (ICD-9-CM 340 or ICD-10-CM G35) within one year or one inpatient hospitalization for MS. This is adapted from a validated study of multiple algorithms used to identify a population of MS patients in a Canadian administrative claims database [17]. There was no exclusion for comorbid conditions. The index date was the first observed medical claim in the database with a diagnosis of MS, resulting in a cohort of newly diagnosed patients. Patients were required to have at least 365 days of continuous enrollment in the database prior to and following their first diagnosis of MS, with no evidence of a prior treatment with a DMT.

### Data source

The analysis was executed in Optum© De-Identified Clinformatics® Data Mart Database, a US-based administrative claims database. Includes 84 million members with private health insurance, who are fully insured in commercial plans or in administrative services only and Medicare Advantage (Medicare Advantage Prescription Drug coverage. The population is representative of US commercial claims patients (0–65 years old) with some Medicare (65+ years old). At the time of this study data were available from May 31, 2000 through June 30, 2019.

The database contains data from adjudicated health insurance claims and health plan enrollment information. Data elements included were outpatient pharmacy dispensing claims (coded with National Drug Codes), inpatient and outpatient medical claims which provide diagnosis codes (coded in ICD-9-CM or ICD-10-CM) associated with a visit. The use of the Optum claims database was reviewed by the New England Institutional Review Board (IRB) and was determined to be exempt from broad IRB approval, as this research project did not involve human subjects research.

### DMT treatment patterns

Treatment patterns included all FDA approved DMTs during the patient identification period except for siponimod and cladribine (both approved March 2019) due to an insufficient number of records in the database (most recently available data through June 2019). The DMTs included were glatiramer acetate, dimethyl fumarate, interferon beta-1a, interferon beta-1b, peginterferon beta-1a, fingolimod, teriflunomide, ocrelizumab, natalizumab, rituximab, alemtuzumab, daclizumab, mitoxantrone. Treatment sequences were captured from index date through all available follow-up, a minimum of 1 year. The term “treatment line” is used to describe the sequence of medication and combinations of medications received by patients during this time. Use of a specific medication was captured at the first instance and not counted again in later lines of therapy – for example an individual filling glatiramer, switching to natalizumab, and then moving back to glatiramer would only be captured as switching from glatiramer to natalizumab. The time a patient was continuously receiving medication is referred to as a drug era. Drug eras were calculated as the time from the first fill for a drug in a medication until discontinuation of that medication, allowing for gaps of up to 30 days beyond the days supply of a prescription (Appendix Fig. 1). Combination therapy with multiple medications was defined as having at least 30 days of overlap in drug eras of more than one treatment. A fill for a medication following discontinuation of a previous drug or with fewer than 30 days of overlap was considered a switch.

### Patient characteristics and comorbidities

Patient characteristics captured include demographics (age, gender, health plan type) on the index date, the Charlson comorbidity index for the year preceding the index date, and individual comorbid conditions during the 1 year following (and including) the index date. Comorbid conditions required just a single diagnosis and were defined using Systematized Nomenclature of Medicine - Clinical Terms (SNOMED CT) classification system. The SNOMED CT classification allows mapping of various diagnostic languages across more than 80 countries, including, for example, ICD-9-CM, ICD-10-CM, and Read codes, to a single standardized set of concepts, and is used by the common data model leveraged for this study [18, 19], described in the next section.

### Common data model

Data from all the database were mapped to standard concepts according to the Observational Medical Outcomes Partnership (OMOP) Common Data Model v5.0 [20] and the treatment sequence analysis was performed within the Observational Health Data Sciences and Informatics (or OHDSI, pronounced “Odyssey”) framework.

## Results

We identified 5691 patients diagnosed with incident MS and meeting the inclusion criteria. Patients were 52.8 years old on average and 73% were female (Table 1). Medicare plans covered 41.2% of patients, while the remainder were on commercially insured plans. All patients were followed for a minimum of 1 year with a median follow-up of 879 days (2.4 years).

**Table 1:** Patient demographics and follow-up observation time

Characteristic	Value
Age (years), Mean (SD)	52.8 (16.1)
Age: < 20	1.5%
Age: 20–24	2.7%
Age: 25–34	10.1%
Age: 35–44	17.9%
Age: 45–54	21.3%
Age: 55–64	19.0%
Age: 65–74	18.9%
Age: 75–84	6.6%
Age: 85+	2.1%
Gender: Female	73.0%
Insurance type: Medicare	41.2%
Charlson comorbidity index score, Mean (SD)	1.77 (2.55)
Follow-up time	
Proportion of patients with at least x days of follow-up	
≥ 365 days	100.0%
≥ 730 days	62.6%
≥ 1095 days	36.3%
Mean follow-up (days)	973
Std. deviation	441
Median follow-up (days)	879
Received a DMT any time during follow-up	35.0%
Time (days) from MS diagnosis to receiving DMT, Mean (SD)	169 (258)

The most common comorbid conditions identified during the one-year post-index period, which included the index date, are found in Table 2. Hypertension was the most common comorbidity following a diagnosis of MS (41.3%), followed by hyperlipidemia (29.0%) and vitamin D deficiency (28.0)%, which has been tied to both risk of MS and disease activity [21]. Other commonly prevalent conditions include symptomatic conditions such as pain, weakness and fatigue, mental health comorbidities including anxiety disorder and depression, and metabolic conditions including obesity and type 2 diabetes, among many others.

**Table 2:** Top 25 comorbidities (SNOMED) diagnosed during the year following the first diagnosis of MS

Condition	Proportion
Essential hypertension	41.3%
Hyperlipidemia	29.0%
Vitamin D deficiency	28.0%
Headache	21.9%
Low back pain	21.8%
Anxiety disorder	21.1%
Muscle weakness	17.5%
Urinary tract infectious disease	17.3%
Chest pain	17.0%
Neck pain	16.7%
Fatigue	16.4%
Dizziness and giddiness	15.8%
Dyspnea	15.2%
Gastroesophageal reflux disease without esophagitis	15.2%
Asthenia	14.9%
Cough	14.7%
Major depression, single episode	14.6%
Paresthesia	13.9%
Hypothyroidism	13.4%
Abdominal pain	13.1%
Type 2 diabetes mellitus without complication	12.2%
Chronic pain	11.7%
Obesity	11.7%
Anemia	11.6%
Cervical spondylosis without myelopathy	11.2%

From the nearly six thousand patients diagnosed with MS identified in our study, 1994 (35.0%) received a DMT at any time during follow-up. The average time from the initial MS diagnosis to the first DMT treatment was 169 days. Of those receiving a DMT, 28.2% (*n* = 563) went on to receive a second line of therapy, 5.8% (*n* = 115) received a third, and 0.9% (*n* = 18) went on to a fourth line during their observed follow-up. Use of more than one DMT simultaneously was uncommon, occurring in just 1.8% of all treated patients. Glatiramer acetate and dimethyl fumarate were by far the most common first-line treatments received accounting for nearly 62% of patients receiving a DMT (Table 3). The distribution of treatments becomes more diverse after the first line with dimethyl fumarate, teriflunomide, ocrelizumab, fingolimod, and natalizumab all accounting for more than 10% of patients receiving a second DMT. And for patients receiving at least three distinct lines of DMT, the monoclonal antibody ocrelizumab is the most common treatment choice.

**Table 3:** Top 10 most common DMTs during each of the first four lines of therapy

Treatment line	Medication	Patient count	Rank in treatment line	% of patients in treatment line
1 (*n* = 1994)	Glatiramer	693	1	34.8%
	Dimethyl fumarate	539	2	27.0%
	Interferon beta-1a	152	3	7.6%
	Fingolimod	151	4	7.6%
	Teriflunomide	142	5	7.1%
	Ocrelizumab	120	6	6.0%
	Natalizumab	85	7	4.3%
	Interferon beta-1b	30	8	1.5%
	Peginterferon beta-1a	30	8	1.5%
	Rituximab	28	10	1.4%
2 (n = 563)	Dimethyl fumarate	117	1	20.8%
	Teriflunomide	88	2	15.6%
	Ocrelizumab	83	3	14.7%
	Fingolimod	82	4	14.6%
	Natalizumab	58	5	10.3%
	Glatiramer	55	6	9.8%
	Interferon beta-1a	31	7	5.5%
	Interferon beta-1b	10	8	1.8%
	Peginterferon beta-1a	8	9	1.4%
	Glatiramer & Dimethyl fumarate	7	10	1.2%
3 (n = 115)	Ocrelizumab	23	1	20.0%
	Dimethyl fumarate	21	2	18.3%
	Natalizumab	16	3	13.9%
	Teriflunomide	16	4	13.9%
	Fingolimod	11	5	9.6%
	Glatiramer	10	6	8.7%
	Interferon beta-1a	8	7	7.0%
	Alemtuzumab	4	8	3.5%
	Peginterferon beta-1a	2	9	1.7%
	Rituximab	2	9	1.7%
4 (n = 18)	Ocrelizumab	7	1	38.9%
	Alemtuzumab	3	2	16.7%
	Natalizumab	3	3	16.7%
	Glatiramer	2	4	11.1%
	Dimethyl fumarate	1	5	5.6%
	Dimethyl fumarate & Interferon beta-1b	1	5	5.6%
	Teriflunomide	1	5	5.6%

**Fig. 1:**
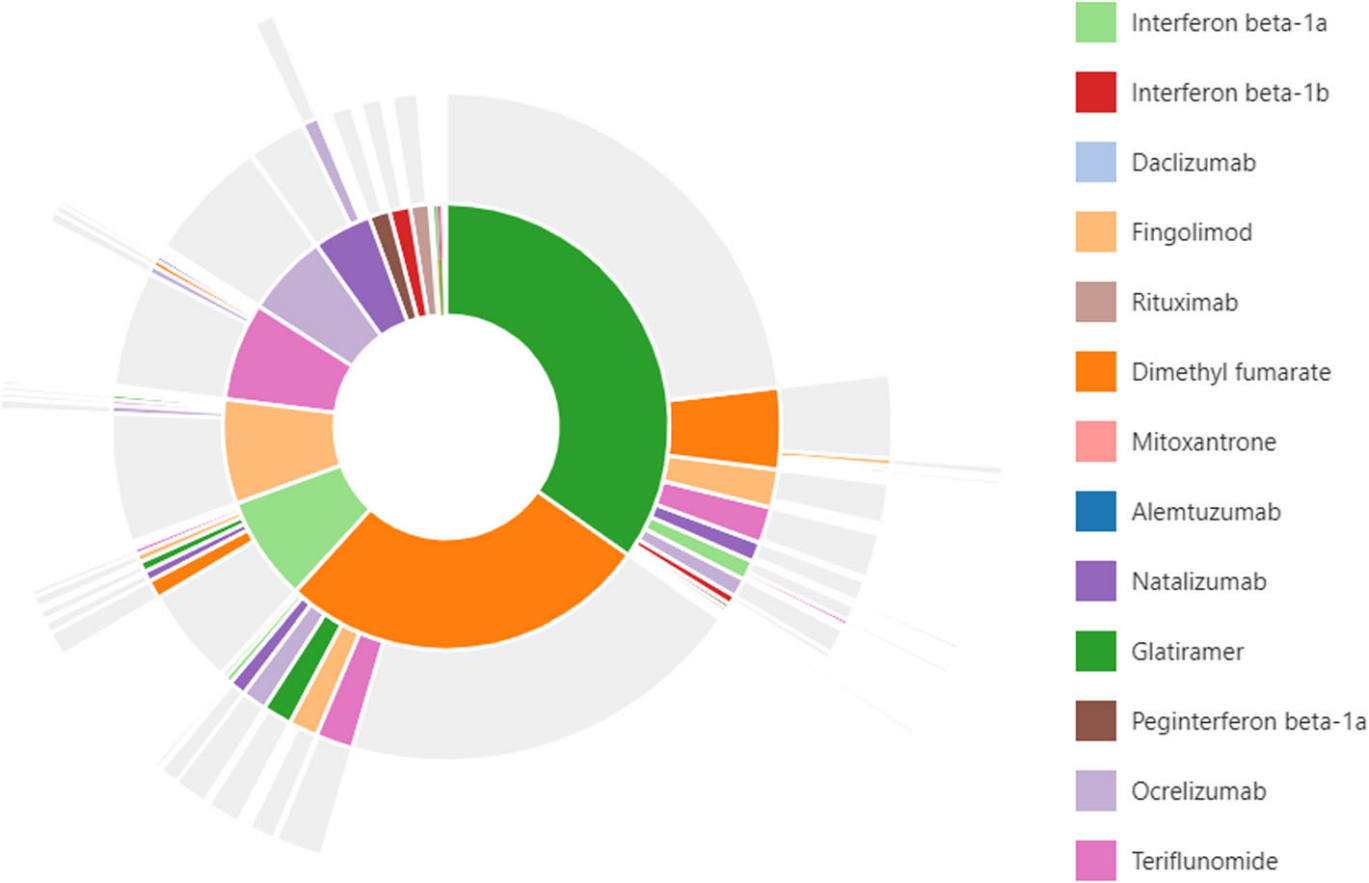
Sunburst of treatment patterns starting with first line (inner-most donut) to fourth line (outer slices). Each color represents a distinct medication, and each layer represents a new treatment line and illustrates the sequence in which patients received different therapies; for example the large green piece in the middle indicates first-line glatiramer use, and the dark orange slice on the next outer ring adjacent to the green indicates a switch from glatiramer to dimethyl fumarate. Slices that have multiple colors indicate combination therapy with more than one medication. Slices in grey indicate no additional medication was taken

Sequences of treatments are shown in the sunburst figure presented in Fig. 1. This figure illustrates that glatiramer acetate and dimethyl fumarate are the most common first-line therapies, accounting for more than half of initial DMT use. Moving from the inner ring to outer rings, we can see that most individuals receive only their first DMT. Second treatment varies according to what the first treatment received was. The most common treatment change for those initiating glatiramer is a switch to dimethyl fumarate, but for those who initiate treatment on dimethyl fumarate, the most common switch is to teriflunomide, while fingolimod, glatiramer, and ocrelizumab make up a similar share of second DMT choice. Treatments were also dichotomized into categories of those that are typically used as first line treatments (Group A: interferons, dimethyl fumarate, teriflunomide, and glatiramer) versus all others (Group B: fingolimod, natalizumab, ocrelizumab, and others). More than half of patients (53%) who filled a second therapy made a switch within Group A, while an additional 35% made a vertical switch from Group A to Group B. The remainder either switched within Group B (8%) or switched from Group B to Group A (4%).

## Discussion

This work fills a knowledge gap on the treatment sequences of DMTs in patients newly diagnosed with MS. Much of the prior research examining treatment patterns has focused on persistence of DMT therapy received [11–15], but there is lack of examining the whole picture of DMT use, including what DMTs are first used and what patients switch to if receiving more than one DMT. This study provides information on the first treatment change as well as up to two additional treatment changes over a period averaging about 2.5 years.

The MS treatment landscape is complex and quickly evolving [22]. During the first decade of the millennium there was a significant shift in the way MS patients were being treated [16], and our research shows that in the decade following it has dramatically changed again. Further change is likely to occur over the next 10 years as there has been a recent influx of new therapies.

Current guidelines published by the American Academy of Neurology (AAN) recommend use of DMT therapies after physician consultation with the patient and include statements on starting, switching, and discontinuing DMTs [23]. Similarly, the European Committee of Treatment and Research in Multiple Sclerosis (ECTRIMS) and the European Academy of Neurology (EAN) recommend early treatment with DMTs to patients with active RRMS [24]. This analysis shows that one-third of patients newly diagnosed with MS went on to receive a DMT during their entire observation. Even when considering that a small portion of the population may not have been RRMS patients, either because they have another form of MS or they were incorrectly classified in the claims data, there still appears to be a gap between what the recommended treatment course is and what is happening in the real world. The claims data lacks details on disease severity, and guidelines have suggested that patients with established “benign MS” may have their disease activity monitored rather than treated with a DMT [25]. The claims data also lacks details on reasons for treatment decisions, and so it is unknown what considerations were discussed before determining whether to initiate use of a DMT or not. Follow-up in this study is also limited (median 2.4 years) and all patients were newly diagnosed in the past 5 years; it may be that many MS patients do not receive a DMT immediately following their initial diagnosis, but as their disease progresses and as they experience more relapses and MRI activity they are more likely to receive a DMT. Additionally, symptomatic treatments, such as use of corticosteroids, were not a focus of this study and are not captured here.

The high prevalence of comorbid conditions across a spectrum of different disease areas illustrates how MS can have a large effect on a patient’s overall health. Diagnoses of pain, weakness, and fatigue illustrate the physical toll the disease presents, while an increase in the prevalence of anxiety and depression exemplify the impact the disease has on the mental health of its patients. And increased rates of cardiovascular disease, diabetes, and obesity may be reflective of the downstream consequences of having limited mobility due to MS, though some of these conditions could be reflective of the older age of this population, independent of MS. The common comorbidities highlighted in this study are consistent with prior research [26], which have been shown to have a significant impact on patients’ quality of life [27, 28], and are associated with DMT use [29]. It should be noted that many comorbidities, especially symptoms that may not require a visit to a physician, may be underrepresented in claims databases. For example, there were 16.4% of patients diagnosed with fatigue in this study, whereas most literature reports a much higher rate. For instance, the North American Research Committee on Multiple Sclerosis (NARCOMS) registry of more than 25,000 patients found that 81% reported some fatigue within the first year after disease onset [30].

The mean age of patients in this study was more than 50 years old, notably higher than the often-reported onset primarily occurring between 20 and 40 years of age [24, 31]. However, prior work utilizing claims data that includes both commercially insured and Medicare enrollees has also reported a mean age more than 42 [12] and 52 years old [32]. Furthermore, an examination of the UK CPRD data [33], which includes observational data over the entire lifespan of individuals, found that the mean age of patients newly diagnosed with MS was 42 years. It is possible that our study included a higher proportion of progressive MS than the estimated 15% of patients with MS diagnosed initially with the primary progressive form of the disease [34]. Primary progressive MS is more common in older adults and this could explain a portion of the lack of DMT use, as the only approved treatment for progressive MS during this study period was ocrelizumab. Unfortunately, there are no ICD-9-CM or ICD-10-CM specific to MS subtypes, and therefore we cannot differentiate RRMS from progressive types and all newly diagnosed MS patients were included. This study represents a real-world commercially insured population in the US, and it appears that a larger proportion of older patients are being diagnosed with MS than what is widely quoted. Because there is only a single ICD-9-CM and one ICD-10-CM code that are used to diagnosis MS, it minimizes the uncertainty of whether the correct diagnosis codes were included for capturing MS patients.. Because the algorithm used to identify MS patients used two outpatient visits or a primary diagnosis during an inpatient hospitalization, there is less of a chance of falsely classifying a patient as having MS due to a rule-out or misdiagnosis that may happen if only requiring a single diagnosis. But, as is shown in a prior validation of various MS definitions using claims data [17], there is a tradeoff between sensitivity and specificity depending on the number of diagnoses required and over what time period they are captured. The validated algorithm most similar to ours required 4 diagnoses within 2 years (we used 2 diagnoses within 1 year) or an inpatient diagnosis, and had a positive predictive value of 82.0%, sensitivity of 88.3%, and specificity of 99.9%. Administrative claims data also lacks details on many clinical measures such as MRI activity (e.g., lesions and brain volume), disability measures (e.g., the Expanded Disability Status Scale), or relapses. This analysis includes patients enrolled in private health insurance and includes both commercial (employer) plans and Medicare Advantage, and results may not be generalizable to other populations, such as those on public health insurance plans (Medicare, Medicaid) or the uninsured.

## Conclusions

Use of DMTs is present in approximately one-third of newly diagnosed MS patients and is largely driven by use of glatiramer acetate and dimethyl fumarate. Only a quarter of those treated go on to receive more than one DMT during follow-up. The comorbid burden of MS patients is significant and diverse, effecting the physical and mental well-being of individuals.

## Supplementary information

**Additional file 1.** APPENDIX Fig. 1. Study design illustration for drug eras, switching and combination therapy classification. A) Drug eras are illustrated assuming a 30-day supply for each medication fill and allowing for a 30-day gap between the end of supply and the next fill. The drug era ends if another fill is not received within this gap. (B) If drug eras of two classes overlap at least 30 days (Drug Class B and Drug Class C) then it is classified as combination therapy, otherwise it is a switch between two classes (Drug Class A to Drug Class B).

## Data Availability

The datasets used and/or analyzed during the current study are available from the corresponding author on reasonable request.

## Abbreviations

AAN: American Academy of Neurology
CNS: Central nervous system
DMT: Disease modifying therapy
EAN: European Academy of Neurology
ECTRIMS: European Committee of Treatment and Research in Multiple Sclerosis
ICD-9-CM: International Classification of Diseases, Ninth Revision, Clinical Modification
ICD-10-CM: International Classification of Diseases, Tenth Revision, Clinical Modification
MS: Multiple sclerosis
OMOP: Observational Medical Outcomes Partnership
OHDSI: Observational Health Data Sciences and Informatics
RRMS: Relapsing remitting multiple sclerosis
SNOMED CT: Systematized Nomenclature of Medicine - Clinical Terms
SPMS: Secondary progressive multiple sclerosis
