# Supplementary material for: Treatment patterns and comorbid burden of patients newly diagnosed with multiple sclerosis in the United States
Source: BMC Neurol. 2020 Aug 11;20:296. doi: 10.1186/s12883-020-01882-2 (PMC7418327; doi:10.1186/s12883-020-01882-2)
Supplement: Supplementary file 1 — Additional file 1. APPENDIX Fig. 1. Study design illustration for drug eras, switching and combination therapy classification. A) Drug eras are illustrated assuming a 30-day supply for each medication fill and allowing for a 30-day gap between the end of supply and the next fill. The drug era ends if another fill is not received within this gap. (B) If drug eras of two classes overlap at least 30 days (Drug Class B and Drug Class C) then it is classified as combination therapy, otherwise it is a switch between two classes (Drug Class A to Drug Class B). [file 12883_2020_1882_MOESM1_ESM.docx]

**APPENDIX FIGURE 1. Study design illustration for drug eras, switching and combination therapy classification.** A) Drug eras are illustrated assuming a 30-day supply for each medication fill and allowing for a 30-day gap between the end of supply and the next fill. The drug era ends if another fill is not received within this gap. (B) If drug eras of two classes overlap at least 30 days (Drug Class B and Drug Class C) then it is classified as combination therapy, otherwise it is a switch between two classes (Drug Class A to Drug Class B).

**
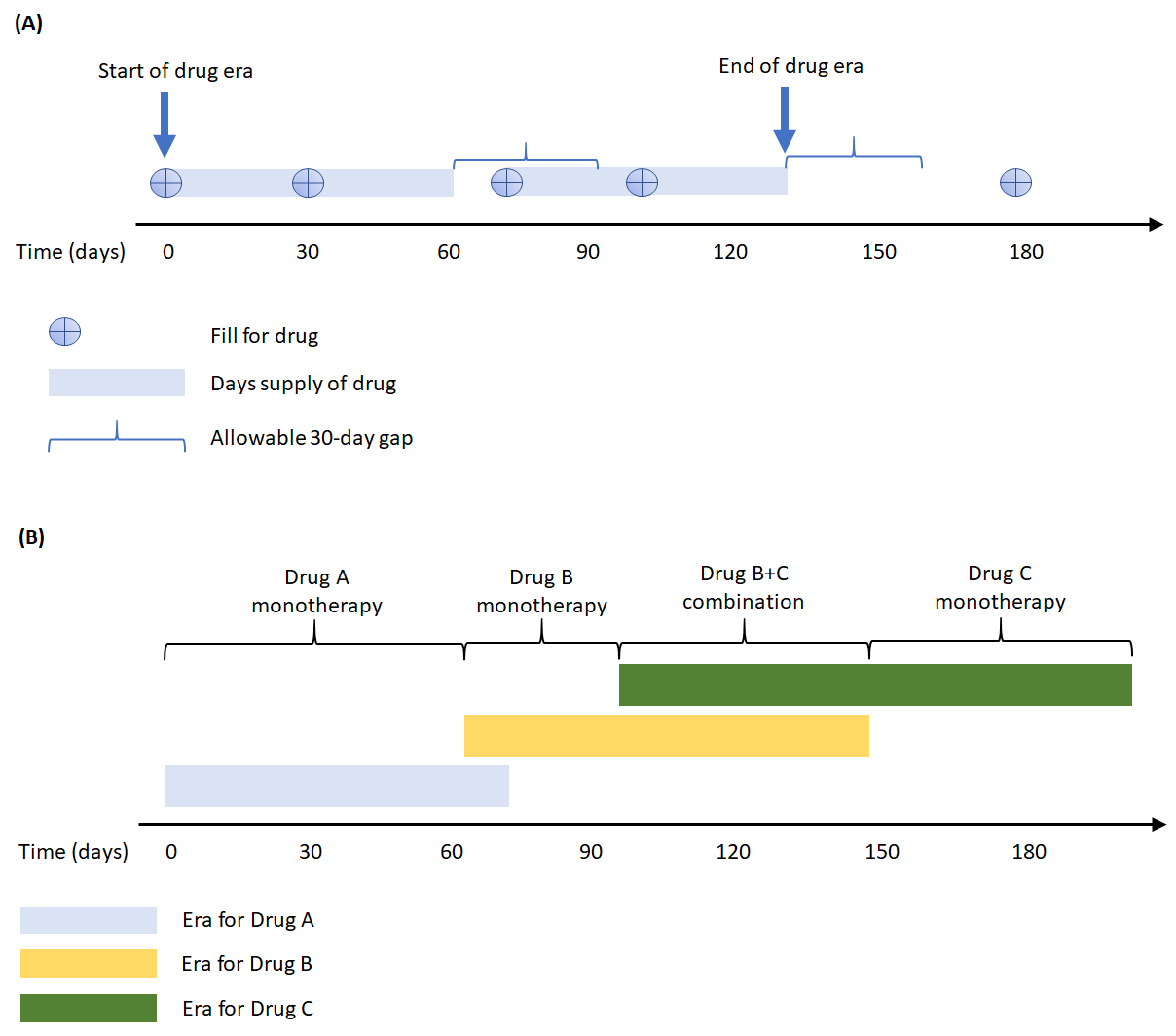
**
